# Treatment of Hemorrhagic Vocal Polyps by Pulsed Dye Laser-Assisted Laryngomicrosurgery

**DOI:** 10.1155/2015/820654

**Published:** 2015-10-18

**Authors:** Hyung Kwon Byeon, Ji Hyuk Han, Byeong Il Choi, Hye Jin Hwang, Ji-Hoon Kim, Hong-Shik Choi

**Affiliations:** ^1^Department of Otorhinolaryngology, Yonsei University College of Medicine, Gangnam Severance Hospital, 211 Eonju-ro, Gangnam-gu, Seoul 135-720, Republic of Korea; ^2^Institute of Logopedics and Phoniatrics, Yonsei University College of Medicine, Seoul, Republic of Korea

## Abstract

*Objective*. Conventional surgical techniques of laryngomicrosurgery (LMS) on hemorrhagic vocal polyps are often difficult due to obscuration of the surgical field by inadvertent bleeding from the lesion, and there are often significant amounts of mucosal epithelium loss. Here, we introduce our surgical technique using pulsed dye laser (PDL), which can effectively resect the polyp with vocal fold mucosa preservation. *Methods*. Patients who were diagnosed with hemorrhagic vocal polyp and who were surgically managed using PDL from March 2013 to October 2014 were retrospectively reviewed. Preoperative and postoperative clinical outcomes and surgical findings were evaluated. *Results*. A total of 39 patients were treated with PDL-assisted enucleation LMS. The average age was 43.7 years (range 20–73), and there were 20 males and 19 females (17 professional voice users). In all cases, the hemorrhagic polyp was successfully enucleated after application of PDL, thereby preserving the overlying epithelium. Postoperative voice outcomes were favorable with clear preservation of the vocal fold mucosal wave. *Conclusion*. PDL-assisted enucleation LMS for the treatment of hemorrhagic vocal polyps can be a safe and effective surgical technique. It can be considered a promising treatment option for hemorrhagic vocal polyps.

## 1. Introduction

Hemorrhagic vocal polyps are the most commonly encountered benign lesions of the vocal fold. They are more often found in men than in women and are usually located on the anterior two-thirds of the vocal fold [1, 2]. The main causative mechanism for hemorrhagic vocal polyps is acute or chronic mechanical phonotrauma to the microvasculature of the superficial layer of lamina propria (SLP). The hyperfunctional glottal sound production causes shearing stress, leading to capillary bleeding within the SLP. The resulting malformed neovascularized mass can be easily ruptured, leading to hemorrhagic events within the vocal fold proper, which in turn hinder normal propagation of vocal fold mucosal waves and cause increased vocal effort. This creates further phonotrauma to the vocal fold, which eventually exacerbates the evolving polyp. The main involvement site is the SLP, and the overlying mucosal epithelium is typically normal; however, the latter may also be involved, having either a keratotic appearance from increased frictional force between the vocal folds or a mucosa-overabundant appearance from the volumetric expansion off the polyp within the SLP [3–5].

Conventionally, hemorrhagic vocal polyps are surgically resected either by the amputation technique using cold microinstruments or CO_2_ laser or by the subepithelial microflap resection technique [6]. The amputation technique results in a significant amount of epithelial loss, especially if the polyp is broad-based, and postoperative vocal outcomes can be undesirable. Additionally, although CO_2_ laser can achieve optimal hemostasis and improved precision, it frequently results in mucosal scarring [7]. The transmitted heat energy from the CO_2_ laser inevitably affects both the epithelium and the SLP, which can lead to fibrosis and increased mucosal stiffness, thereby ultimately resulting in deterioration of voice quality. In the subepithelial microflap resection technique, although the overlying epithelium is raised by epinephrine-saline infusion, the operation can often be cumbersome due to a certain amount of inevitable bleeding owing to the nature of the hemorrhagic polyp. The 585 nm pulsed dye laser (PDL) is an angiolytic laser causing selective photothermolysis. The chromophore of the PDL is oxyhemoglobin, resulting in the laser inducing photocoagulation of microvascular lesions with minimal damage to the surrounding normal tissue. Based on our recent successful reports of PDL application in treating sulcus vocalis and glottic leukoplakia [8, 9], we have managed to use PDL in surgically removing hemorrhagic vocal polyps. This study aims to report our surgical technique, PDL-assisted enucleation laryngomicrosurgery (LMS), which can effectively resect the polyp with vocal fold mucosa preservation.

## 2. Methods

### 2.1. Patients

This study was conducted on 39 patients who were admitted and received PDL-assisted enucleation LMS under general anesthesia for hemorrhagic vocal polyps despite persistent conservative treatment at the Department of Otorhinolaryngology, Gangnam Severance Hospital, from March 2013 to October 2014. Inclusion criteria of the patients were as follows: (1) pathologically proven hemorrhagic vocal polyps and (2) evident hemorrhagic vocal polyps on preoperative videostrobolaryngoscopy (Laryngograph Ltd., London, UK). Exclusion criteria were as follows: (1) simple, nonhemorrhagic vocal polyps and (2) other accompanying structural vocal fold abnormalities. All patients provided written informed consent before the surgery, and the Institutional Review Board of Yonsei University College of Medicine approved this retrospective study.

### 2.2. Surgical Technique

**Figure 1:**
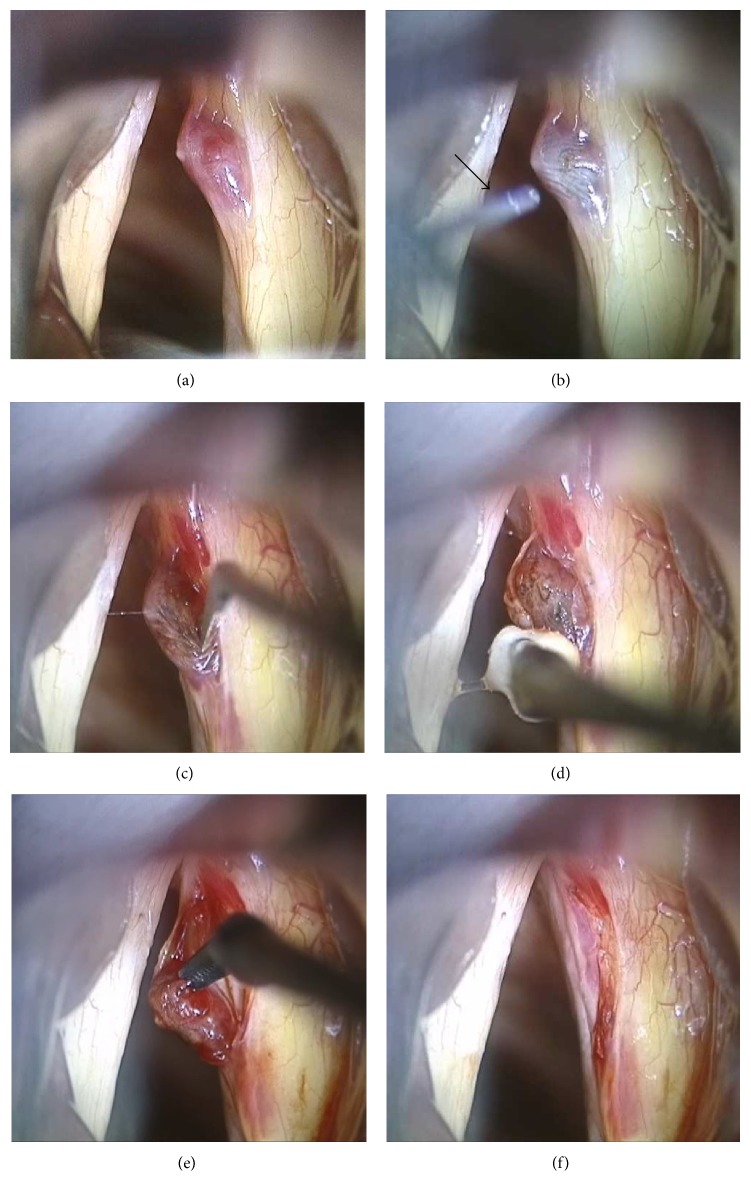
Intraoperative view of pulsed dye laser- (PDL-) assisted enucleation laryngomicrosurgery. (a) After suspension laryngoscopy under general anesthesia, a hemorrhagic vocal polyp is noted on the right vocal fold. (b) The PDL is delivered by a 0.6 mm fiber (arrow), which is held directly over the surface of the hemorrhagic polyp. The treated portion of the vocal fold can be confirmed by the blanching change of the epithelium. (c) After the PDL application, a longitudinal incision is made at the overlying epithelium. (d) The epithelium is easily peeled off from the lesion and opened using a small cotton ball mounted on microforceps. (e) After careful dissection with appropriate microinstruments, the hemorrhagic polyp is easily enucleated out using microcurved alligator forceps. (f) The remaining epithelium is repositioned after removal of the lesion.

All operations were performed solely by the senior author (Hong-Shik Choi). Each step of the operation is illustrated (Figure 1). After clearly exposing the lesion under suspension laryngoscopy, the 585 nm PDL (Cynosure Inc., Chelmsford, MA, USA) was applied above the polyp, though without direct contact (450 *μ*s pulse width, 2.0 J/pulse maximum output, and 2 Hz repetition rate). The PDL was delivered via a 0.6 mm flexible fiber with a constantly delivered energy of 0.75 J/pulse, and an average of 20 pulses (range 8–49) was applied for each vocal polyp. After confirming the blanching change of the overlying epithelium, an incision was made, and, with careful dissection, the epithelium could be easily “peeled away” from the polyp lesion. Further dissection was performed with microinstruments, and eventually only the polyp was extracted, leaving the overlying epithelium unharmed. The epithelium was repositioned carefully to cover the surgical defect, and the operation was completed. Following the operation, the patients were prescribed strict voice rest for 7 to 10 days and were also counseled on vocal hygiene and behavioral vocal changes.

### 2.3. Clinical Outcome Assessment

For evaluation of treatment outcomes, all patients were given voice assessments and laryngeal stroboscopic examinations before and two months after the operation. Voice analysis including aerodynamic measures (Phonatory Aerodynamic System; KayPENTAX, Montvale, NJ, USA), acoustic analysis, and electroglottographic analysis (EGG) (Lx speech studio program; Laryngograph Ltd., London, UK) was conducted, and voice handicap index (VHI) was examined. Auditory perceptual judgment was carried out in a recorded sample called “autumn” [10], which was approximately two minutes in length, with the use of the GRBAS scale (G, grade; R, roughness; B, breathiness; A, asthenia; S, strain). The recorded samples were evaluated in a blinded manner by two speech pathologists. The point scale of each category was 0 to 3 points, and the mean of two values each obtained from two speech pathologists was used for the analysis. The patients were followed up for a minimum of 4 months to a maximum of 9 months until it was considered that there was no need for further followup.

The demographic, clinicopathological, and treatment characteristics were retrospectively reviewed and analyzed. The following voice parameters were evaluated: maximum phonation time (MPT), mean airflow rate (MFR), subglottic pressure (Psub), closed quotient (CQ), irregularity of frequency percentage (CFx), irregularity of amplitude percentage (CAx), average fundamental frequency (F0), noise-to-harmonic ratio (NHR), jitter percentage, and shimmer percentage. Preoperative and postoperative results of each voice parameter were statistically compared using paired *t*-test with SPSS 18.0 software for Windows (SPSS, Chicago, IL, USA), with statistical significance defined as a *P* value less than 0.05.

## 3. Results

The mean age of the studied population was 43.7 years (range 20–73 years), and there were 20 males and 19 females. Among these patients, 17 (43.6%) were professional voice users. The hemorrhagic vocal polyp was located on the right vocal fold in 17 patients, on the left in 19, and on both in 3 patients.

**Figure 2:**
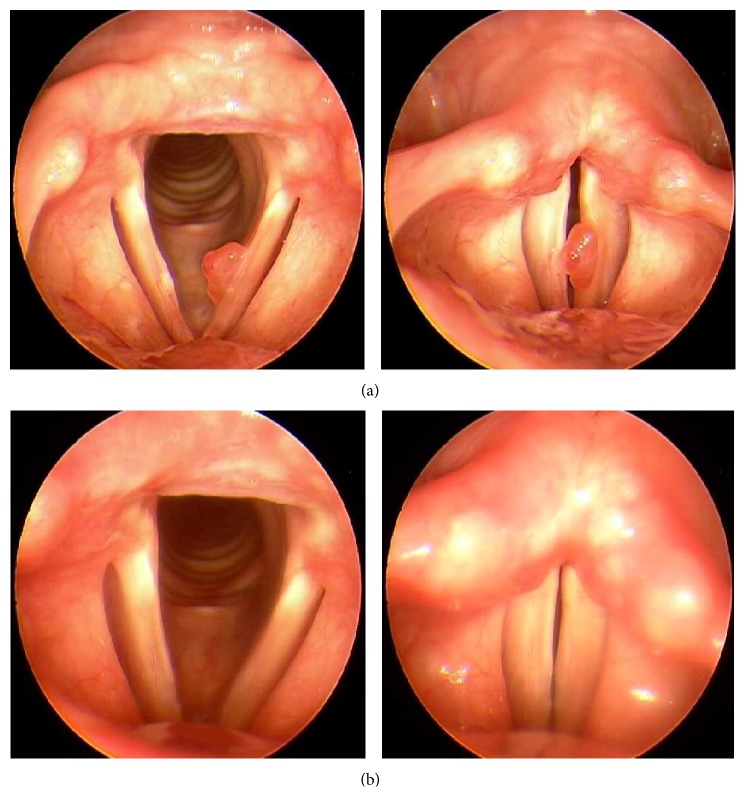
(a) Preoperative laryngeal stroboscopic images. A typical hemorrhagic vocal polyp with a sessile base is noted on the left vocal fold. (b) Laryngeal stroboscopic images taken two months after the operation. Notice the preservation of the mucosal wave.

All operations were successfully accomplished without any significant intraoperative complications. There was almost no bleeding during each operation, and after removal of the polyp, there were minimal amounts of mucosal loss (Figure 1). Preservation of the mucosal wave was noted in all cases on postoperative stroboscopic examination (Figure 2). During the follow-up period of the patients, there was no recurrence of the polyp or any other laryngeal lesion development.

Changes of each voice parameter after the operation are summarized in Table 1. VHI score and GRBAS scale scores from auditory perceptual judgment showed significant improvements after the operation. Other objective measures of voice analysis showed a general improvement, but only significant differences were noted in MFR and jitter percentage.

## 4. Discussion

The normal vocal fold is composed of five layers: the epithelium, the three layers of the lamina propria (superficial, intermediate, and deep), and the thyroarytenoid muscle. According to Hirano's cover-body theory of vocal fold vibration, the epithelium and the SLP constitute the “cover,” the vocalis muscle acts as the “body,” and the intermediate and deep layers of the lamina propria, which make up the vocal ligament, are the “transition” layer [11]. The cover layer is considered important, as it is mainly involved in the normal mucosal wave vibration of the vocal folds. The basement membrane zone (BMZ) is a collection of extracellular matrix (ECM) that attaches and secures the overlying epithelium and the SLP underneath. The BMZ is further divided into two distinct layers, the superficial lamina lucida (LL) and the deep lamina densa (LD). The LL connects the basal epithelium by hemidesmosomes, and the LD is attached to the SLP by anchoring fibers consisting of collagen type VII. The LL and the LD are bound by anchoring filaments made of collagen type IV and fibronectin [12]. Hemorrhagic vocal polyps are known to develop from phonotrauma such as severe voice abuse or misuse, and these benign vocal fold lesions can lead to acute or persistent dysphonia or hoarseness. Its main pathogenesis is microvascular trauma within the BMZ and the SLP. Although some hemorrhagic vocal polyps may resolve spontaneously with minimal or conservative treatment [13], surgery is the standard treatment of choice for persistent polyps after voice therapy and observation.

A PDL beam with a 585 nm wavelength is selectively absorbed by hemoglobin, and the energy from the laser beam penetrates the superficial epithelium without causing damage yet results in intravascular coagulation of subepithelial microvasculature. The laser was initially adopted in dermatologic clinics to treat port-wine stains, telangiectasias, and any other cutaneous vascular lesions. Recently, the PDL has generated substantial interest in the field of phonosurgery for treating various laryngeal lesions. Since the initial attempt of McMillan et al. of applying PDL in treating laryngeal papillomas [14, 15], there have been numerous reports of PDL application in the management of leukoplakia, glottic dysplasia, granuloma, and vascular abnormalities of the vocal folds [16–19]. The utility of the PDL has been further verified with the treatment of glottic hyperkeratosis and sulcus vocalis by Choi et al. [8, 9].

The photoangiolytic effect of the PDL can greatly facilitate the operation, not only from improved hemostasis, but also due to the induration effect of the lesion. From the results of this study, the hemostasis could control the natural bleeding tendency of the hemorrhagic vocal polyp, thereby avoiding any troublesome obstruction of surgical view with bleeding. Additionally, we observed that the lesion became relatively hardened due to the photocoagulation caused by the PDL, which further aided in the manipulation and dissection of the polyp. Essentially, there are three fundamental effects of laser on living tissue: photoacoustic, photothermal, and photochemical effects. According to the ultrastructural evaluation made by Ayala et al., the photoacoustic and photothermal effects of the PDL create a cleavage plane, specifically between the LL and LD of the BMZ [20]; thus, the PDL treatment would cause the mucosal epithelium to separate and elevate above the lesion within the polyp-containing vocal fold. Therefore, from this effect, not only could the epithelium be easily “peeled off” from the surface of the polyp, achieving enhanced precision of cold instrumental dissection and selective extraction of the polyp, but the all-important cover layer of the vocal fold could also be preserved with appropriate wound healing, allowing the reestablishment of normal mucosal wave vibration and phonation after the surgery. From the results of the laryngeal stroboscopic examinations (Figure 2), favorable postresection mucosal pliability and glottal closure could be verified in all cases, and voice assessment results showed general improvement of voice quality following the surgery (Table 1). There has been an earlier report by Zeitels et al. of treating the hemorrhagic vocal polyp by an angiolytic laser after raising a subepithelial microflap with saline solution infusion [19]. Our specific technique goes one step further, exploiting not only the photocoagulative effect of PDL but also the cleavage plane formation effect created by the laser. The application of PDL as an initial step of the surgery omits the necessity of subepithelial infusion.

Given that PDL is easy to use and is a safe and efficacious tool for laryngeal lesions, it has been incorporated as a minimally invasive technique for use in office-based procedures [17, 21]. The so-called “awake” PDL technique for treating vocal polyps does not require resection; however, it may lead to repeated procedures and a longer postoperative recovery period. It may also present certain limitations due to the constant movements of the larynx in conscious patients. The procedure described in the present study, however, is performed under general anesthesia and can be considered as one particular method of LMS utilizing a PDL as an effective surgical tool. It may be a useful, alternative surgical technique to the conventional surgical resection of hemorrhagic vocal polyps by subepithelial microflap elevation followed by subepithelial infusion of saline and epinephrine as initially reported by Hochman and Zeitels [6]. Although further investigation with a longer period of follow-up and a larger volume of patients may be required to establish the feasibility of the PDL-assisted enucleation LMS technique, this procedure was found to be effective in removing hemorrhagic polyps and was easy to perform. It has also shown promising postoperative results with a decreased period of healing.

## 5. Conclusion

The PDL-assisted enucleation LMS technique can be easily and effectively performed to treat hemorrhagic vocal polyps indicated for surgery. The mucosal wave of the vocal fold is well preserved as postoperative healing is improved, resulting in good voice outcomes. This surgical technique may also prove to be a valuable treatment for various laryngeal lesions.

## Figures and Tables

**Table 1:** Voice quality improvement after the operation.

	Preoperative	Postoperative	*P* value
Aerodynamic measures, mean (SD)			
MPT (sec)	12.43 (5.73)	14.35 (5.50)	0.084
MFR (L/sec)	0.16 (0.13)	0.11 (0.09)	0.005^*^
Psub (cmH_2_0)	6.80 (2.08)	6.23 (2.28)	0.287

EGG analysis, mean (SD)			
CQ (%)	42.42 (5.45)	42.91 (8.06)	0.728
CFx (%)	12.50 (10.89)	9.54 (6.83)	0.197
CAx (%)	6.86 (3.41)	6.35 (4.60)	0.515

Acoustic analysis, mean (SD)			
*F*0 (Hz)	158.62 (38.14)	157.21 (41.61)	0.630
NHR	0.15 (0.03)	0.14 (0.02)	0.121
Jitter (%)	2.33 (1.58)	1.50 (0.87)	0.006^*^
Shimmer (%)	4.55 (2.92)	3.70 (1.61)	0.134

Patient-perceived satisfaction, mean (SD)			
VHI score	40.82 (28.62)	13.96 (13.50)	<0.001^*^

Auditory perceptual judgment, mean (SD)			
G	1.32 (0.54)	0.63 (0.46)	<0.001^*^
R	0.57 (0.40)	0.26 (0.38)	<0.001^*^
B	0.86 (0.48)	0.37 (0.32)	<0.001^*^
A	0.03 (0.19)	0 (0)	0.326
S	0.32 (0.36)	0.18 (0.24)	0.004^*^

IQR: interquartile range; MPT: maximum phonation time; MFR: mean airflow rate; Psub: subglottic pressure; CQ: closed quotient; CFx: irregularity of frequency; CAx: irregularity of amplitude; *F*0: average fundamental frequency; NHR: noise-to-harmonic ratio; VHI: voice handicap index; G: grade; R: roughness; B: breathiness; A: asthenia; S: strain.

∗ refers to the values that are statistically significant (*P* value < 0.05).
